# Temporal and Fine-Grained Pedestrian Action Recognition on Driving Recorder Database

**DOI:** 10.3390/s18020627

**Published:** 2018-02-20

**Authors:** Hirokatsu Kataoka, Yutaka Satoh, Yoshimitsu Aoki, Shoko Oikawa, Yasuhiro Matsui

**Affiliations:** 1 National Institute of Advanced Industrial Science and Technology (AIST), Tsukuba 305-8560, Japan; yu.satou@aist.go.jp; 2 Department of Electronics and Electrical Engineering, Keio University, Yokohama 223-8522, Japan; aoki@elec.keio.ac.jp; 3 Tokyo Metropolitan University, Tokyo 192-0364, Japan; shoko_o@hotmail.com; 4 National Traffic Safety and Environment Laboratory, Tokyo 182-0012, Japan; ymatsui@ntsel.go.jp

**Keywords:** fine-grained pedestrian action recognition, two-stream convnets, driving recorder, advanced driver-assistance systems (ADAS)

## Abstract

The paper presents an emerging issue of fine-grained pedestrian action recognition that induces an advanced pre-crush safety to estimate a pedestrian intention in advance. The fine-grained pedestrian actions include visually slight differences (e.g., walking straight and crossing), which are difficult to distinguish from each other. It is believed that the fine-grained action recognition induces a pedestrian intention estimation for a helpful advanced driver-assistance systems (ADAS). The following difficulties have been studied to achieve a fine-grained and accurate pedestrian action recognition: (i) In order to analyze the fine-grained motion of a pedestrian appearance in the vehicle-mounted drive recorder, a method to describe subtle change of motion characteristics occurring in a short time is necessary; (ii) even when the background moves greatly due to the driving of the vehicle, it is necessary to detect changes in subtle motion of the pedestrian; (iii) the collection of large-scale fine-grained actions is very difficult, and therefore a relatively small database should be focused. We find out how to learn an effective recognition model with only a small-scale database. Here, we have thoroughly evaluated several types of configurations to explore an effective approach in fine-grained pedestrian action recognition without a large-scale database. Moreover, two different datasets have been collected in order to raise the issue. Finally, our proposal attained 91.01% on National Traffic Science and Environment Laboratory database (NTSEL) and 53.23% on the near-miss driving recorder database (NDRDB). The paper has improved +8.28% and +6.53% from baseline two-stream fusion convnets.

## 1. Introduction

Understanding a pedestrian intention is an important work in the recent ADAS and self-driving cars. In an urgent situation, a couple of seconds that is generated by a pedestrian intention estimation could be critical to avoid a collision.

**Figure 1:**
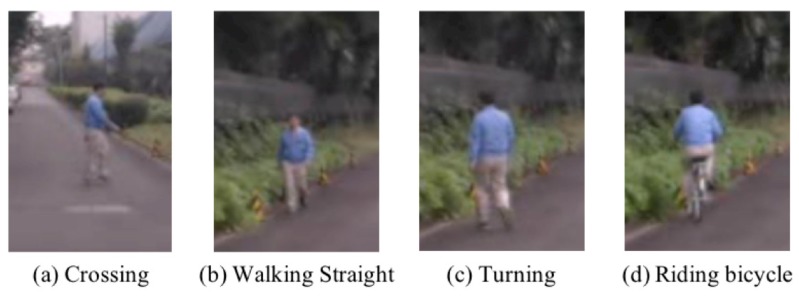
Fine-grained pedestrian actions on the self-collected databases: (**a**) crossing; (**b**) walking straight; (**c**) turning; and (**d**) riding a bicycle. Fine-grained pedestrian action recognition should be an issue in safety systems that have a recognition problem with distinguishing different actions between subtle changes. To improve the recent safety systems such as advanced driver assistance systems (ADAS) and self-driving cars, the concept is very important because a pedestrian intention can be estimated in advance.

At first, the research of pedestrian analysis was studied in vision-based localization in traffic safety systems [1,2]. The research topic has focused on feature descriptors, classifiers and training strategies from an Red, Green and Blue (RGB)-image input. The performance of pedestrian localization has been rapidly increased due to the burden of experiments about pedestrian localization methods. The noteworthy stream was so-called deep neural networks (DNN), which can automatically learn an effective feature at each recognition task. The DNN performs better than conventional approaches [3,4,5] in recognition tasks such as image recognition [6,7]. The DNN methods have been applied into intelligent transport systems (ITS) studies, which include the pedestrian localization [8,9]. We believe that the pedestrian study should be shifted to the next step. Therefore, the paper poses a new problem to the pedestrian study in the ITS field, namely “fine-grained pedestrian action recognition”, which is to distinguish different actions between subtle changes. The fine-grained pedestrian action recognition, such as walking straight into turning (see Figure 1), is quite important to estimate an intention for more advanced safety systems.

There are three difficulties with achieving the concept of fine-grained pedestrian action recognition. The first difficulty is to capture a small but meaningful change in walking pedestrian actions. The problem should be considered with a sophisticated feature representation. In the second, a vehicle-mounted camera is always moving and the background is cluttered. We should extract a suitable feature in a focused area by excluding moving/cluttered background areas. The third difficulty is data collection. The collection of intention change is very difficult due to the rarity of turning action in actual driving. We should construct a feature extraction with relatively small video data.

The paper proposes a novel concept of fine-grained pedestrian action recognition for intention estimation in a safety system. Two databases have been collected by dividing two scenarios into experimental and practical scenes. The experimental dataset contains fine-grained pedestrian actions in a static background, and the practical dataset includes fine-grained actions from a driving recorder. The paper also proposes a convnet-based descriptor that contains a couple of modifications to adapt problem-specified difficulties. In addition to the baseline architecture, a feature enhancement and parameter adaptation has been implemented. The experimental section shows the effectiveness of database and system configuration, and demonstrates pedestrian intention recognition with fine-grained pedestrian action recognition.

The paper is organized as follows. Section 2 summarizes related work. The databases and proposed approach are presented in Section 3 and Section 4, respectively. The experimental results are presented and discussed in Section 5. Finally, Section 6 summarizes the paper.

## 2. Related Work

### 2.1. Pedestrian Detection

Since Dalal et al. presented the histograms of oriented gradients (HOG) [3], pedestrian detection has been an active topic in computer vision. The HOG method has been improved into the Co-occurrence HOG (CoHOG) [10] and Extended CoHOG (ECoHOG) [11]. The CoHOG is known as a high-standard detection approach for pedestrian detection by representing edge pair [10]. Moreover, the ECoHOG replaced edge-pair by gradient magnitude in order to represent strength of curves and lines [11]. Dollar et al. followed the gradient-based features; they confirmed that their integral channel feature (ICF) [12] has resulted in faster and more accurate descriptions of pedestrians.

Due to the great success of convolutional neural networks (CNN), image classification has greatly improved since the era of codeword vectors [13,14,15]. An outstanding result was obtained by AlexNet [16] in the ImageNet large-scale visual recognition challenge 2012 (ILSVRC 2012), and this is a large impact on the use of deep neural networks in computer vision. Recent models, such as the Visual Geometry Group Net (VGGNet) [6], GoogLeNet [17] and residual networks (ResNet) [7], are known as deeper architectures. Above the line of CNN, we have obtained a sophisticated detection algorithm, Region-based CNN (R-CNN) [18]. The R-CNN is constructed of two phases, namely generating object proposal and category classification. Although the detection algorithm performs better than the conventional detection methods such as HOG and integral channel features (ICF), the original R-CNN is absolutely slow in terms of processing speed (47 s/image). Therefore, the faster algorithm is proposed in Fast/Faster R-CNN [19,20]. The recent algorithms (e.g., Single-shot multibox detector (SSD) [21], you only look once (YOLO) [22,23]) have been improved toward a real-time processing. Zhang et al. [8] provided both a sophisticated model and dataset in pedestrian detection. They claimed that a more sophisticated annotation was required to improve a detection. The well-organized works [24,25] have been extensively studied and compared with human-level detection, and it is considered to be the state-of-the-art method for pedestrian detection. Unlike the pedestrian detection with RGB-input, Dalal et al. [26] and González et al. [27] have applied a temporal channel with optical flows.

The performance of pedestrian detection is being closer to the human-level performance. According to the line, we believe that the pedestrian study should be shifted to the next step. Our proposal is to conduct a pedestrian intention estimation based on the fine-grained action recognition. At first, space-time representation is described including a couple of works for fine-grained action recognition. Then, the recent traffic databases are listed.

### 2.2. Space-Time Representation

Space-time interest points (STIPs) have been the primary focus for action recognition [28]. In a STIP, the time *t* space is added to the x,y spatial domain. The most important approach is that of dense trajectories (DT) [29], which track densely sampled feature points. In addition, Wang et al. proposed the IDT [30], which estimates the camera motion in order to remove the detection-based noise; it also incorporates a Fisher vector [15].

Recently, temporal models with CNN have been proposed [31,32,33]. Tran [31] proposed a convolution model for xyt maps that is based on the RGB sequence. The 3D convolutional networks (C3D) approach directly captures the temporal features in an image sequence. Another approach, two-stream CNN, is a well-organized algorithm that captures the temporal feature of an image sequence [32]. The integration of the spatial and temporal streams allows us to effectively enhance the representation of motion. We thus better understand how the spatial information relates to the temporal feature.

### 2.3. Traffic Database

Several practical databases for pedestrian detection and autonomous driving have been proposed in the past decade. Representatives include the INRIA person dataset [3], the Caltech pedestrian dataset [34], and the KITTI dataset [35]. Dalal et al. [3] created the INRIA person dataset. These were important contributions to solving the problem of pedestrian detection. Dollar et al. followed their work, and pursued the problem of pedestrian detection by using the Caltech pedestrian dataset [34,36]. Their detailed analysis was beneficial for improving the descriptors, classifier, and model, removing several difficulties regarding analysis of the benchmark.

The KITTI has been used to set meaningful vision problems for autonomous vehicles [35]; these include problems in stereo vision, optical flow, visual odometry, semantic segmentation, 2D/3D object detection, and 2D/3D tracking. For stereo and optical flow, the problems were updated in 2015 [37]. Thanks to sophisticated approaches, such as fully convolutional networks (FCN) [38] and R-CNN, there has been improved performance for solving these problems using the KITTI benchmark dataset. In addition, a manner of geometry allows us to improve the rate of object detection [39] and the optical flow [40] not only in stereo [41]. In semantic segmentation, the method extracts knowledge about dense connections, and this can be used with a graphical model [8,42].

Against the databases, our proposed database provides a problem toward a pedestrian intention recognition based on the fine-grained pedestrian action recognition. There is an urgent need for a collection of pedestrian videos to implement and evaluate the significant recognition work.

## 3. Self-Collected Databases

The section presents two self-collected databases about fine-grained pedestrian action recognition, National Traffic Science and Environment Laboratory database (NTSEL) and near-miss driving recorder database (NDRDB). Based on the definition of fine-grained recognition (Fine-grained categorization lies in the continuum between basic level categorization (frog vs. piano) and identification of individuals (face recognition, biometrics). The visual distinctions between similar categories are often quite subtle. See [43]), the four pedestrian actions are defined; *walking*, *crossing*, *turning*, and *riding a bicycle* (Figure 1). Intuitively, the pedestrian action categories are divided into different walking directions seen from an in-vehicle camera. Moreover, *riding a bicycle*, which is a confusing category by appearance, is added in the databases. We considered the two different scenarios with (i) a simple and static background for a pure performance test with a space-time representation (NTSEL), and (ii) dynamic and cluttered backgrounds to evaluate a performance in a practical situation (NDRDB). The summary of databases is shown in Table 1. The database description is described as follows.

### 3.1. NTSEL Database (NTSEL)

To evaluate a performance of space-time representation by excluding a background effect, we have experimentally collected traffic videos with four different fine-grained actions in the outside of the laboratory. The video database contains 100 videos in total and these are equally divided (25 videos) per category. The video database is captured in a simple and static background; therefore, we basically analyze how effective the space-time representation is on the NTSEL. The driving recorder is attached and the videos are captured from a static vehicle. In the database, three actors walked and rode a bicycle in front of the driving recorder. The distance is from 3.0 to 20.0 m. The *turning* is occurred at the 5.0, 10.0, 15.0 and 20.0 m distances.

### 3.2. Near-Miss Driving Recorder Database (NDRDB)

The society of automotive engineering of Japan (JSAE) is providing the Hiyari–Hatto database, which includes near-miss incidents [44]. The database contains video, GPS (global positioning system) and CAN (controller area network) data. We focused on pedestrian actions to analyze their fine-grained categorization in practical situations. Although the near-miss videos are difficult to collect, 82 videos have been collected in the database. The driving recorders are attached on a moving vehicle; therefore, four fine-grained actions are set—*walking*, *crossing*, *standing* and *riding a bicycle*. The moving camera makes the computer vision difficult with problems such as motion blur, and relative motion between vehicle and pedestrian. The database contains 15 (walking), 43 (crossing), 13 (standing) and 11 (riding a bicycle) videos, respectively.

### 3.3. Difficulties of Self-Collected Databases

By using the collected databases, there are three main difficulties with achieving the concept of fine-grained pedestrian action recognition:

- We should notice small changes in pedestrian actions, namely a meaningful change such as walking straight into turning should be recognized. In many cases, the change occurs in a moment. Therefore, a sophisticated descriptor is preferable to catch a subtle difference.
- An in-vehicle driving recorder is moving depending on the vehicle ego-motion (The NTSEL database does not include moving background. However, the database contains difficulties coming from a cluttered background and fine-grained pedestrian actions.). The fine-grained pedestrian action recognition should be done in a cluttered scene that contains complicated and moving backgrounds.
- The collection of pedestrian fine-grained action is difficult. A large amount of fine-grained pedestrian action data allows us to significantly understand fine-grained pedestrian actions from a vehicle-mounted driving recorder. Although the collection of such data is very difficult due to the rarity of action change such as turning in actual driving, we should treat a feature extraction and learning in a small-scale walking action database, with the aim of improving the avoidance of accidental situations. Therefore, we should consider how to learn about a strong model with a small-scale database.

## 4. Proposed Approach

**Figure 2:**
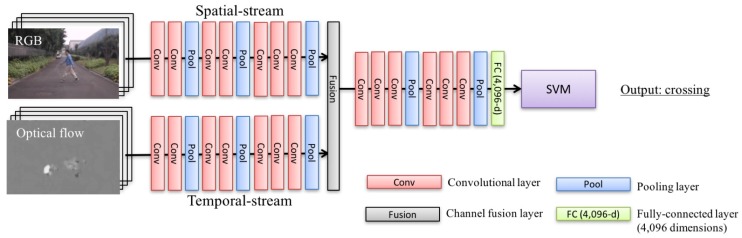
Flowchart of our proposed approach: Proposed architecture for fine-grained pedestrian action recognition. We assign two-stream fusion convnets [45] originally proposed by Feichtenhofer et al. The conventional work operates channel-sum with two different convolutional maps in an intermediate layer of spatial- and temporal-stream. After the channel fusion layer (“fusion” in the architecture), we add several convolutional and pooling layers (conv and pool) in order to generate a strong feature, e.g., subtle difference in walking pedestrian. In the classification step, we employ deep convolutional activation features (DeCAF; the first fully-connected layer (FC) with 4096-d vector) to converge the small-scale database by combining with support vector machines (SVM) [46]. Two-stream fusion convnets and DeCAF + SVM are trained with a training-set on self-collected databases.

Based on the two-stream fusion convnets [45], we have constructed an improved classifier in fine-grained pedestrian action recognition. The section shows the proposed architecture and a couple of improvements as follows:

**Architecture.** The proposed approach is shown in Figure 2. The basemodel, two-stream fusion convnets [45], supplies a better representation than conventional two-stream convnets [32] with fused convolutional maps and additional convolutions. In the fusion layer (“Fusion” in Figure 2), we can get a more sophisticated representation through several additional convolutions after layer-fusion between RGB-input and flow-input. Intuitively, the multiple modality analysis allows us to extract important features such as small changes of moving area in a pedestrian’s sequence.

The basic architecture consists of two-stream fusion convnets [45] (convolutional maps; *m*), deep convolutional activation features (DeCAF) [46] (vector; *v*), and SVM (category; *c*) from inputs from RGB ($I_{rgb}$) and optical flow ($I_{flow}$).

We begin by calculating convolutional maps *m* for a given videos $I_{rgb}$ and $I_{flow}$ in Equation (1):

(1) $$\begin{array}{ccc} m & = & f(I_{rgb},I_{flow};w), \end{array}$$

where the function *f* outputs convolutional maps, which are parameterized by convolutional kernels *w*. We then convert DeCAF *v* from convolutional maps *m* with linear function in fully-connected layer *g* in Equation (2):

(2) $$\begin{array}{ccc} v & = & g(m). \end{array}$$

Finally, a category *c* is trained with an SVM model.

**DeCAF with fine-tuning architecture.** DeCAF is employed with the first fully-connected (fc) layer, which has 4096 dimensions, since we should train with small-scale databases. The DeCAF is known as an effective technique when there is no large-scale database. In our pre-experiment, a training could not converge an end-to-end training with two-stream fusion convnets. Moreover, the activation feature is improved with self-collected databases from an initial pre-trained UCF101 parameters [47,48] through a fine-tuning training. We verified the effectiveness of fine-tuning in the experimental section. Table 2 shows the with or without fine-tuning with NTSEL and NDRDB databases.

**Unit adaptation.** An fc layer is verified with {128, 256, 512, 1024, 2048, and 4096} units. Basically, the original 4096 units based on an ImageNet model are assigned, but the number of dimensions should be fixed depending on the recognition problem. The above-mentioned fc units are compared in the experimental section. Table 3 describes performance rates with various fc units on NTSEL and NDRDB databases.

## 5. Experiment

The section describes the experimental settings, results and discussion.

### 5.1. Implementation

In the spatial-stream, the input was 224 pixels × 224 pixels × 3 channels. In the temporal-stream, a basic stacked flow [32] was implemented in order to create an input of 224 pixels × 224 pixels × 20 channels. All initial learning rates were set to 0.001, and updating was set to a factor of 0.1 at each 1/2 and 3/4 of total learning epochs. The training is terminated maximum 50 epochs (We assigned a model which achieves the best rate). A high dropout ratio is set in each of the fully-connected layers (0.8–0.9 at each fc connection). The mini-batch size is 32 in the experiment.

We split training/testing sets into NTSEL and NDRDB databases. In NTSEL, we set 15 videos for training and 10 videos for testing. In NDRDB, we set 2/3 for training and others for testing. The training and testing splits are fixed for a fair evaluation.

### 5.2. Exploration Study

We carry out a couple of tunings to improve fine-grained pedestrian action recognition. The fine-tuning, fc units and SVM parameter are adjusted in the section.

**With or without fine-tuning (see Table 2).** The results with fine-tuning on both databases are listed in Table 2. Starting from UCF101 pre-trained model by Wang [47], we fit into our NTSEL and NDRDB databases with fine-tuning training. In the situation, we use general purpose video features without fine-tuning, or the traffic video feature with fine-tuning after extracting vectors of DeCAF. As a result of fine-tuning, with fine-tuning is better than without one (+6.00% on NTSEL, +5.07%). The value describes that the traffic specified feature is effective for the problem of fine-grained pedestrian action recognition. A fine-tuned architecture, DeCAF and SVM make an effective configuration. Hereafter, the configuration is used in this experiment.

**Various fc units (see Table 3).** We confirmed that there is a suitable number of units at each database. Table 3 shows the relationship between #fc-unit and its performance rate. According to the table, it is suitable to use 4096-d (91.01%) on NTSEL and 1024/4096-d (53.23%) on NDRDB. 4096-d on NTSEL and 1024-d on NDRDB are assigned as a tuned parameter.

**Figure 3:**
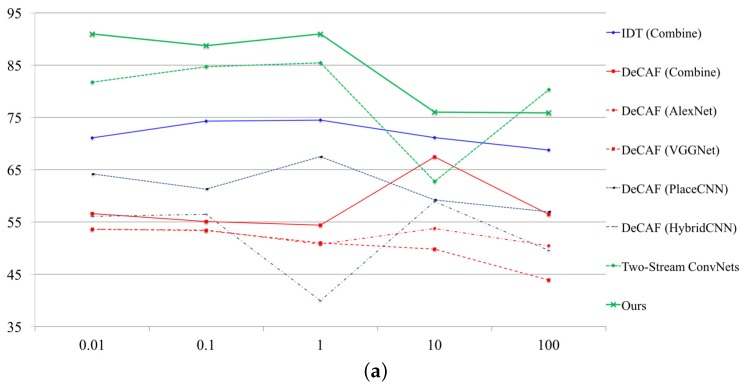

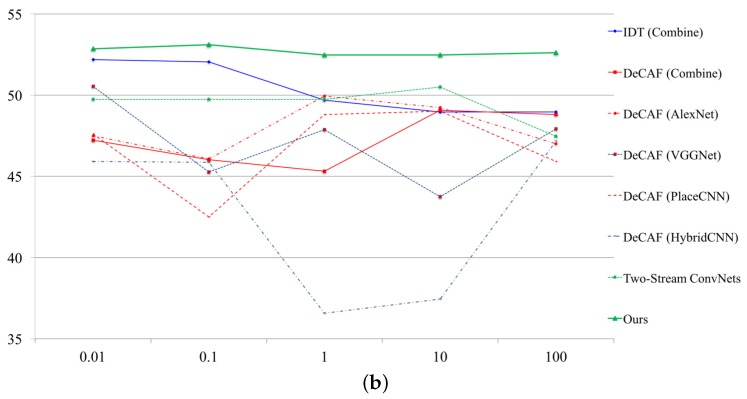
SVM parameter tuning. (**a**) relationship between performance rate and SVM parameter on NTSEL; (**b**) relationship between performance rate and SVM parameter on NDRDB.

**SVM parameter (see Figure 3).** To fix a so-called “c-parameter” in SVM, we tuned several parameters at each database. We adopted the parameters as {0.01, 0.1, 1.0, 10, 100} in Figure 3. In Figure 3, we simultaneously decided the SVM parameters with representative approaches. The parameter depends on the problem, but it is effective for improving the performance rate. Finally, the performance rates have been increased +8.28% on NTSEL (82.73 to 91.01) and 6.53% on NDRDB (46.70 to 53.23).

### 5.3. Comparison of Representative Approaches

We investigated the effectiveness of some motion representations. Here, IDT [30], DeCAF [46] and two-stream convnets [32] are employed. The abstract of the approaches is listed below:

**IDT.** IDT is the de-facto-standard hand-crafted model for video representation. The setting is based on the original implementation. To generate a codeword vector, motion boundary histograms (MBH) (192-d), histograms of optical flow (HOF) (108-d), and HOG (96-d) are captured each time a trajectory is sampled; they are incorporated into a feature vector.

**DeCAF.** Activation features were extracted based on the AlexNet/VGG-16. In the paper, we set fc6 for each CNN architecture. We used ImageNet pre-trained model (ImageNet, ImageNet with VGG-16) [6,16], Places205 pre-trained model (Places205) [49], and ImageNet + Places205 pre-trained model (HybridCNN) [49]. One more model, all combined (ImageNet, ImageNet with VGG-16, Places205, HybridCNN), is applied.

**Two-stream convnets.** We used two-stream CNN based on VGG-16 [47] by Wang et al. The pre-trained model is trained with UCF101, and additional training was performed on self-collected databases.

**Trajectory-pooled deep-convolutional descriptors (TDD) [33].** The TDD is at the intersection of the IDT and two-stream convnets. A large number of trajectories are sampled to access convolutional maps. Here, spatial convolutional maps are used.

The comparison with representative approaches is listed in Table 4. Our proposal achieved the top rate on NTSEL (91.01%) and a competitive rate on NDRDB (52.23%). We have improved two-stream fusion convnets with well-organized parameters. The model assigns both channels of RGB and flow to extract a feature from a minor change in moving area.

Undoubtedly, the approach is effective in NTSEL, which has a static background at each video. In the NTSEL, a major moving area is only pedestrian’s walking; therefore, convnet-based methods with optical flows (ours and two-stream convnets) tend to have high-accuracy on NTSEL. The approaches such as DeCAF (67.48% with Places205) and spatial-stream (69.04%) struggle to enhance the feature for fine-grained pedestrian action recognition. Accuracy was not achieved even if we use a combined DeCAF (67.48%). The flow-based models including IDT can easily catch a suitable feature for classification. The combined IDT with HOG/HOF/MBH achieved 74.52% on NTSEL that is better rate than spatial-convnet; however, the noisy flows on background area are disturbing.

On one hand, fine-grained pedestrian action recognition on NDRDB is difficult due to the moving background, cluttered background and relatively small changes of pedestrian intention. Despite the difficult situation of video recognition, our proposal achieved the second best accuracy on NDRDB. Our two-stream fusion convnets significantly extracts good features by excluding the effects of moving background. We consider the effect is coming from complementation between RGB (texture) and flow (moving area) each other. Hand-crafted IDT recorded a good accuracy with trajectory-based representation. In NDRDB, TDD had the best accuracy with the combination of trajectory-based feature and deep convolutional representation. The method can receive both merits from hand-crafted features and deeply learned representations. The trajectory-based approach picked up background flows, but there are informative features such as ego-motion by a vehicle.

**Discussion.** According to the results on the experiments, the combined streams with spatial (RGB input) and temporal (optical flow input) channels should be implemented in fine-grained pedestrian action recognition. The combined representation simultaneously works with an effective feature extraction with both streams in additional convolutional layers (after fusion-layer in Figure 2), and noisy areas’ exclusion. Moreover, we have improved a performance rate by fine-tuning and DeCAF. In the situation, an end-to-end is not converged with only a small-scale database. There are only $10^{2}$ ($10^{3}$)-order videos (frames) against the pre-training with UCF101 [48], which has 13,220 videos on the database. The other parameter tuning contributed to increasing the performance rates. Finally, we have improved +8.28% and +6.53% from a baseline.

The self-collected databases present an important issue of “fine-grained pedestrian action recognition” in addition to the feature improvement by fine-tuning. Towards a practical-level performance, we should try to improve the accuracy of the problem. The solution will be data collection and model updates from the current configurations.

In the database construction, the limitation of data variation should be treated in the future. For examples, we would like to extend our databases to deploy the pedestrian intention recognition at night. Recently, a couple of databases [50,51] are proposed with various sensors such as far infrared (FIR) and multispectral camera.

To improve the current two-stream fusion convnets, we would like to use hand-crafted knowledge like TDD. Many sampling points allow the model to recover a more sophisticated representation. Moreover, a possible combination is coming from a skeleton-based feature. For example, Fang et al. estimated and quantized pedestrian’s skeletons to extract a stably spatiotemporal vector [52].

### 5.4. Visual Results

**Figure 4:**
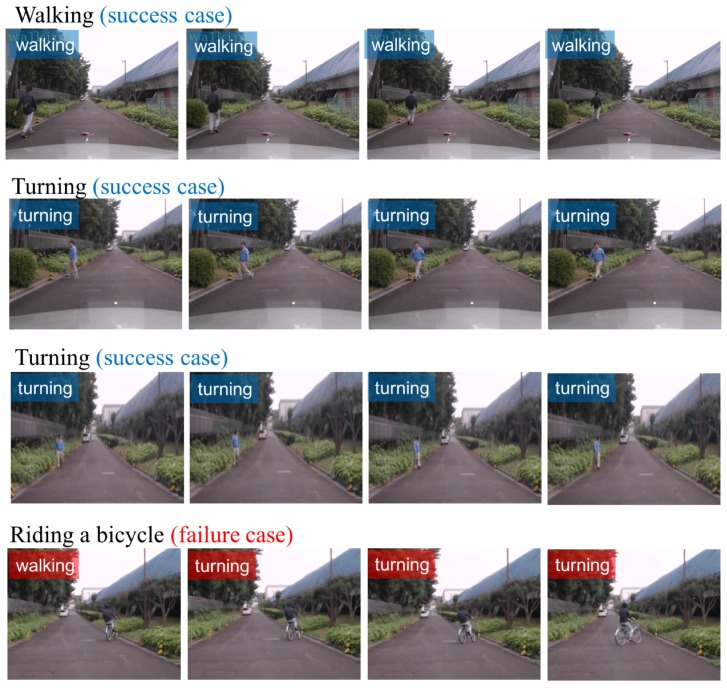
Visual results on NTSEL dataset: the first three lines, there are three success cases as the examples of walking and turnings. The last row shows the failure case in a sequence of a person is riding a bicycle. Especially in the second row, we succeeded with an estimation of pedestrian intention in advance. The turning walking action is important for a safety system.

We list several visual results on NTSEL in Figure 4. There are four different sequences in various actions from a couple of pedestrians. For all pedestrian sequences (the first three rows), the pedestrians are relatively small, but our proposal successfully recognized the action category. Especially in the turning actions (second and third rows), we can estimate a moment in advance. The recognition of turning allows us to predict the next action, e.g., crossing a street and walking straight.

However, in the last row, our proposal corrected riding a bicycle as walking/turning. The second half of the sequence contains a turning action while riding a bicycle, and this is a partially correct answer.

## 6. Conclusions

The paper proposed an important issue, fine-grained pedestrian action recognition for an advanced safety system. The fine-grained pedestrian action recognition allows us to estimate a pedestrian’s intention. We have collected two different databases: NTSEL and near-miss driving recorder database (NDRDB) to provide the recognition problem. The videos included in both databases are captured by vehicle mounted driving recorders. The NTSEL is the simple setting with static background, but there are difficulties such as cluttered background and relatively small pedestrians in a video sequence. The NDRDB is more practical video data by moving in the background. We also proposed a improved convnet-based descriptor as a baseline with two-stream fusion convnets. We added a couple of modifications such as fine-tuning with self-collected databases and a fully-connected unit adaptation. Moreover, we assigned the deep convolutional activation features (DeCAF) since the databases have only $10^{2}$-order size in terms of videos. Due to the results of fine-grained pedestrian action recognition, our proposal achieved the top rate on NTSEL (91.01%) and the second best rate on NDRDB (53.23%). We revealed that the combined streams with RGB and flow inputs are important in order to be highly accurate recognition. Finally, we have increased +8.28% on NTSEL and +6.53% on NDRDB, respectively.

In the future, we need to improve the accuracy of practical settings (e.g., usage of NDRDB) by database collection and model updates.

## Figures and Tables

**Table 1:** Summary of databases.

DB	NTSEL Database; NTSEL	Near-Miss Driving Database; NDRDB
	#Video (#Frame)	#Video (#Frame)
#*Walking*	25 (2648)	15 (515)
#*Crossing*	25 (2726)	43 (1773)
#*Turning*	25 (923)	13 (593)
#*Riding a Bicycle*	25 (1632)	11 (457)
#Total	100 (7929)	82 (3338)

**Table 2:** With or without fine-tuning.

	NTSEL (%)	NDRDB (%)
End-to-End	N/A	N/A
Without fine-tuning (DeCAF)	82.73	46.70
With fine-tuning (DeCAF)	**88.73**	**51.77**

**Table 3:** Various fc units on the self-collected databases.

#Fc-Unit	NTSEL (%)	NDRDB (%)
128	88.58	51.01
256	88.73	48.47
512	89.30	51.49
1024	86.30	**53.23**
2048	89.01	49.87
4096	**91.01**	**53.23**

**Table 4:** The performance rates on the NTSEL & near-miss DR dataset.

Approach	NTSEL (%)	NDRDB (%)
IDT (HOG)	70.18	50.43
IDT (HOF)	64.76	52.05
IDT (MBH)	65.38	49.12
IDT [30]	74.52	52.19
DeCAF (ImageNet) [16]	53.78	49.94
DeCAF (ImageNet with VGG-16) [6]	53.63	50.54
DeCAF (Places205 [49]	67.48	49.02
DeCAF (Hybrid) [49]	58.91	47.17
DeCAF (Combined) [46]	67.44	49.07
Two-stream ConvNets (Spatial)	69.04	48.47
Two-stream ConvNets (Temporal)	64.05	45.93
Two-stream ConvNets [32]	**85.44**	50.50
TDD [33]	68.39	**54.66**
Ours	**91.01**	**53.23**

## Abbreviations

**Important Acronyms**	
DNN	Deep Neural Networks
CNN, convnet(s)	Convolutional Neural Networks
NTSEL	National Traffic Science and Environment Laboratory Database
NDRDB	Near-Miss Driving Recorder Database
Conv	Convolutional Layer
Pool	Pooling Layer
Fusion	Fusion Layer
FC	Fully-Connected Layer
DeCAF	Deep Convolutional Activation Features
SVM	Support Vector Machines
